# Tips and pearls for peripherals nerve blocks for foot surgery: A pictorial essay

**DOI:** 10.1016/j.jatmed.2026.07.003

**Published:** 2026-08-26

**Authors:** Laura Girón-Arango, Carlos Andrés Guzmán-Hernández, Philip Peng

**Affiliations:** a Department of Anesthesiology and Pain Medicine, University of Toronto, Toronto, ON M5G 1E2, Canada; b Department of Anesthesia and Pain Management, Toronto Western Hospital, University Health Network, Toronto, ON M5T 2S8, Canada; c Graphic Designer and Illustrator, Giron Arango Medicine Professional Corporation, Toronto, Canada

**Keywords:** Peripheral nerve blocks, Foot surgery, Ultrasound-guided regional anesthesia, Sonoanatomy, Ankle block

## Abstract

Ultrasound-guided regional anesthesia of the ankle and distal lower extremity is widely used for surgical anesthesia and postoperative analgesia. Success depends on a detailed understanding of nerve anatomy, anatomical variations, and sonoanatomy at clinically relevant levels. This pictorial essay reviews the relevant anatomy, sonoanatomy, and block-specific considerations of the sciatic nerve and its distal branches—including the tibial, common peroneal, sural, and saphenous nerves—with an emphasis on tips and pearls for ultrasound-guided regional anesthesia.

## Introduction

Ankle and foot surgeries are associated with significant postoperative pain.^1^, ^2^ Ultrasound-guided nerve blocks significantly reduce postoperative pain severity and opioid consumption, with effects lasting 24–48 h. In the immediate postoperative period (0–24 h), nerve blocks afford substantial pain reduction compared with general or spinal anesthesia alone and are recommended as first-line analgesic therapy.^3^, ^4^ In our practice, most foot and ankle procedures are ambulatory. It is standard to use an ultrasound-guided ankle block with sedation for foot surgery below the ankle joint, and an ultrasound-guided popliteal sciatic block for analgesia during ankle surgery. Nevertheless, we have found that some novice learners and non-regional anesthesiologists remain uncomfortable with these techniques and rarely incorporate them into routine practice despite the well-known advantages of regional anesthesia for foot and ankle surgery. This discomfort is often related to challenges with image acquisition, accurate identification of neural structures, and needling technique. This pictorial essay serves as an educational resource for anesthesiologists wishing to incorporate these nerve blocks into their practice, providing practical recommendations to enhance image acquisition and needling technique.

## Ankle block

The ankle block targets five distal, predominantly sensory nerves: tibial, saphenous, deep fibular, superficial fibular and sural. This block can be used for surgical procedures involving the foot distal to the ankle joint and is associated with minimal motor blockade, thereby facilitating early ambulation – an important consideration in ambulatory surgery. It can be employed as the sole anesthesic technique for procedures without a tourniquet or with an ankle tourniquet.

Compared with landmark-guided ankle blocks, ultrasound-guided ankle blocks have been shown to increase success rates. However, lower total local anesthetic volumes (mean 16 mL) have been associated with inferior postoperative analgesia compared with conventional volumes (30–40 mL), as reflected in higher postoperative pain scores and increased rescue analgesic requirements.^5^

A high-frequency linear transducer (10–18 MHz) is recommended for most ankle block applications because the target nerves are superficial. Both in-plane and out-of-plane needle approaches can be used, depending on operator preference, target anatomy, and patient characteristics. An in-plane approach allows continuous visualization of the needle shaft and tip.

### Tibial nerve

**Fig. 1:**
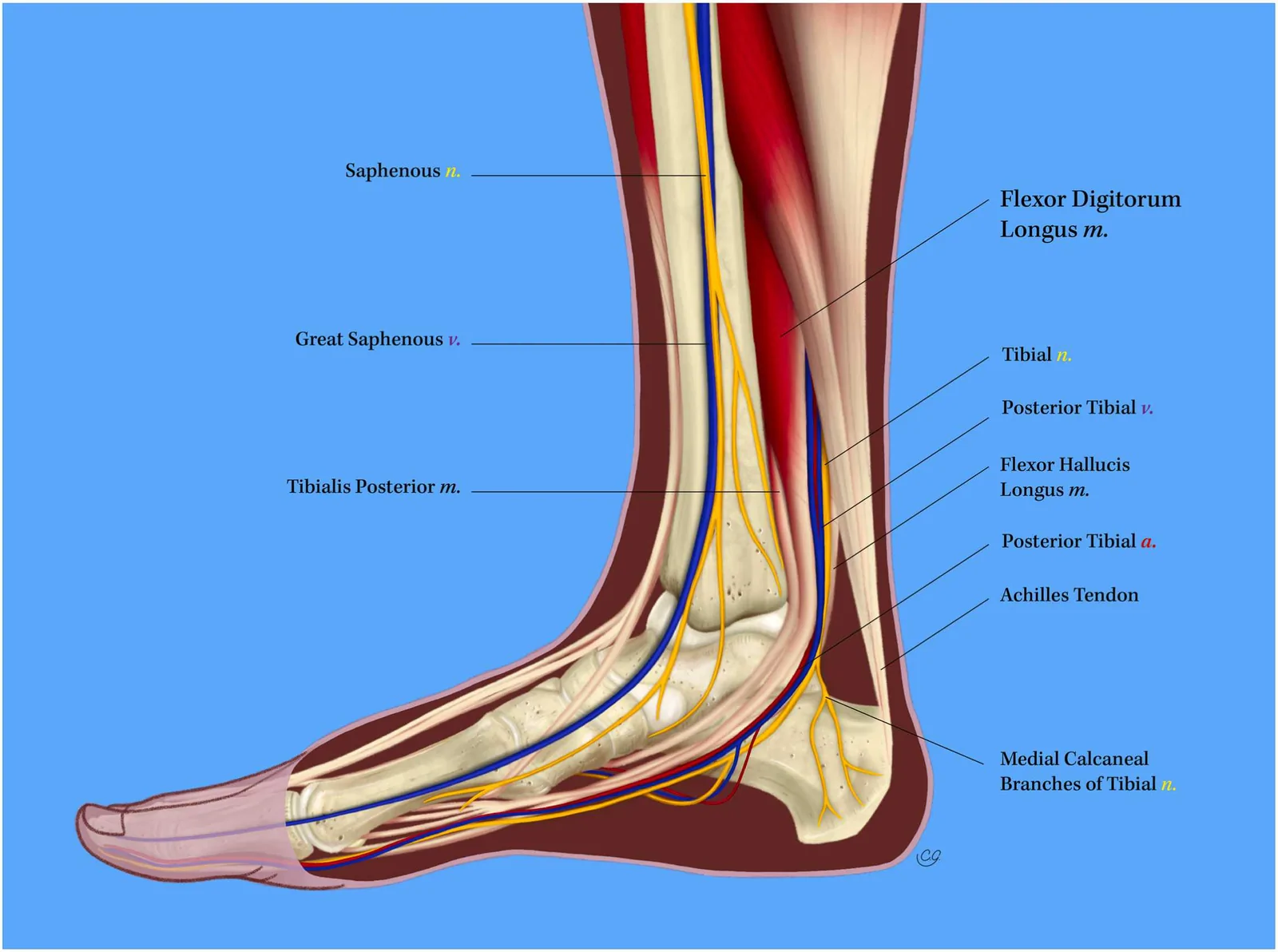
Illustration of the medial aspect of the leg highlighting the saphenous and tibial nerves. a: artery, m: muscle, n: nerve, v: vein. Reproduced with permission from Girón Arango Medicine Professional Corporation.

**Fig. 2:**
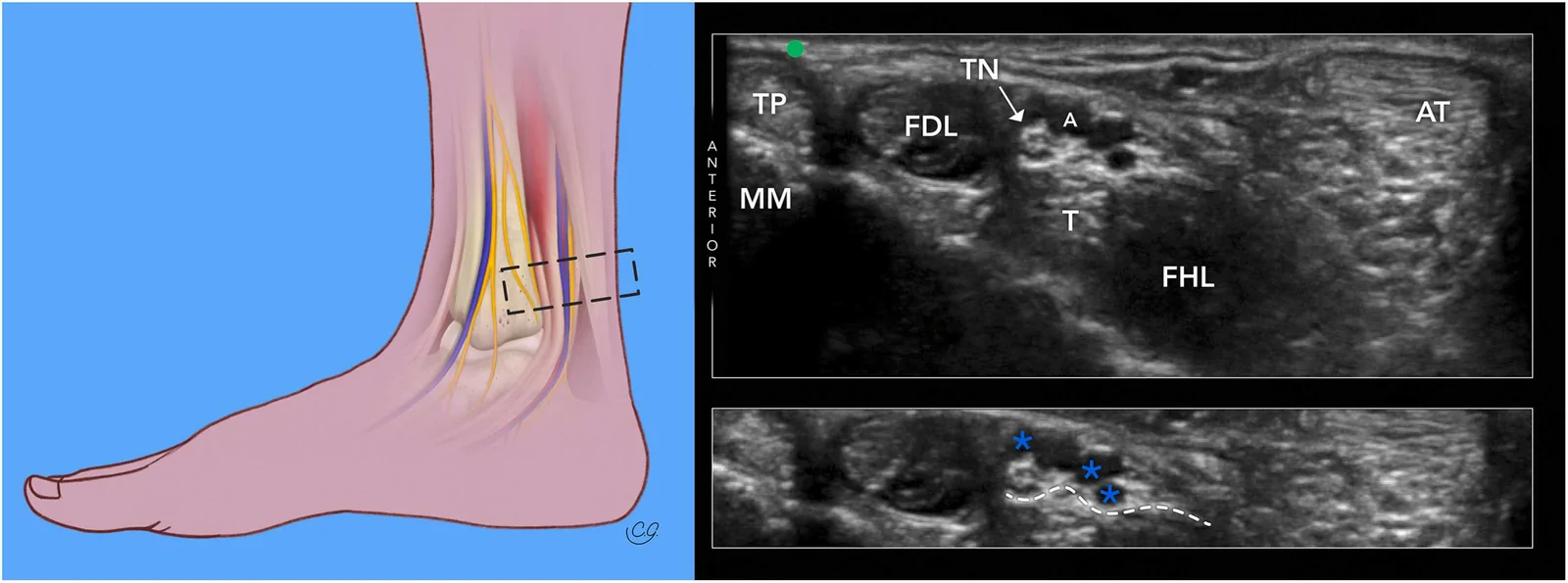
Ultrasound image and corresponding probe position for the tibial nerve block. A: tibial artery, AT: Achilles tendon, FDL: flexor digitorum longus muscle and tendon, TP: tibialis posterior tendon, FHL: flexor hallucis longus muscle and tendon (T), MM: medial malleolus, TN: tibial nerve, dotted white line: flexor hallucis longus fascia, *: veins accompanying the tibial artery. Please note that the flexor retinaculum over the tibial nerve and neurovascular bundle was removed to show the underlying tibial nerve. Reproduced with permission from Girón Arango Medicine Professional Corporation.

The tibial nerve (TN) is the largest branch of the sciatic nerve. After emerging, it travels anterior to the soleus muscle arch and continues into the posterior compartment of the leg with the posterior tibial vessels.^6^ Proximally, the TN gives off branches that innervate the muscles of the posterior compartment of the leg, and distally it has 3 main sensory branches (lateral plantar nerve, medial plantar nerve and calcaneal nerve) that ultimately innervate the plantar aspect of the foot.^6^, ^7^ At the level of the medial malleolus, the tibial nerve is typically located within the tarsal tunnel (medial retromalleolar region), a fibro-osseous canal on the medial aspect of the ankle, posterior to the medial malleolus. It is roofed by the flexor retinaculum and bounded laterally by the medial surfaces of the tibia, talus, and calcaneus.^8^ Distally, the tunnel narrows and blends with the fascia of the abductor hallucis muscle (Fig. 1). From anteromedial to posterolateral, the contents are the tibialis posterior tendon, flexor digitorum longus tendon, posterior tibial artery and vein, tibial nerve, and flexor hallucis longus tendon.^9^ The TN lies anterior to the Achilles tendon and posterior to the posterior tibial vascular bundle. A crucial anatomical landmark for identifying the tibial nerve is the flexor hallucis longus (FHL) tendon, which lies deep to the nerve and serves as a consistent reference point. The fascia overlying the FHL can be used to identify the correct tissue plane, with the neurovascular bundle situated superficial to this structure (Fig. 2). Dynamic movement of the hallux may facilitate identification of the FHL tendon (Video S1). Depending on patient anatomy, needle insertion may be performed using either an in-plane or out-of-plane approach, with local anesthetic deposited adjacent to the nerve along the relevant fascial plane (Video S2). Anatomical variation of the tibial nerve is well described; notably, in approximately 7% of cases, its terminal sensory branches divide proximal to the tarsal tunnel,^10^ with important implications for regional anesthesia, as these branches may be encountered separately when the block is performed at the level of the medial malleolus.

### Deep fibular (peroneal) nerve

**Fig. 3:**
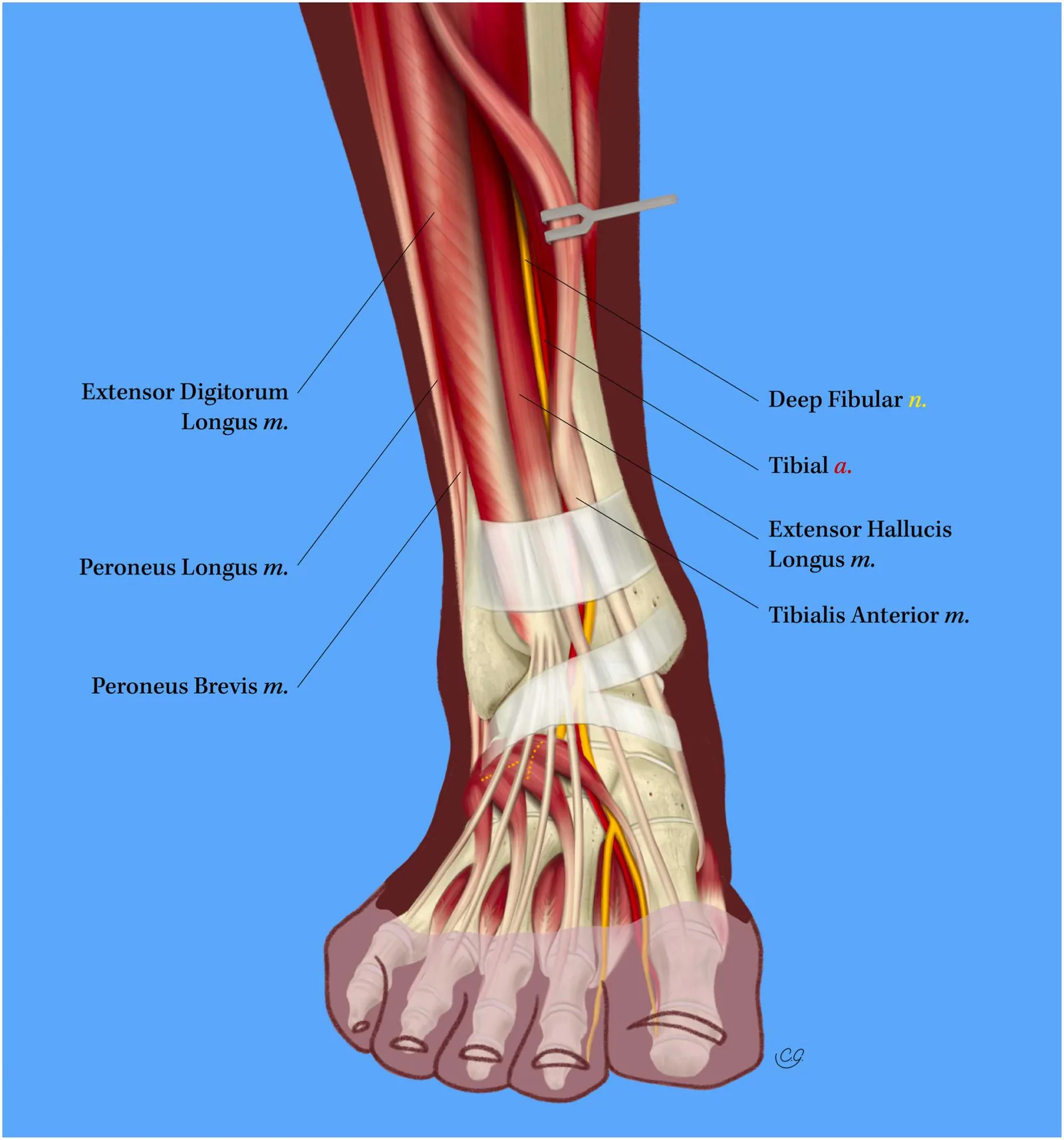
Illustration of the anterior aspect of the leg highlighting the deep fibular nerve. a: artery, m: muscle, n: nerve. Please note that the dorsalis pedis artery and nerve run superficial to the interosseous membrane in the distal leg. Reproduced with permission from Girón Arango Medicine Professional Corporation.

**Fig. 4:**
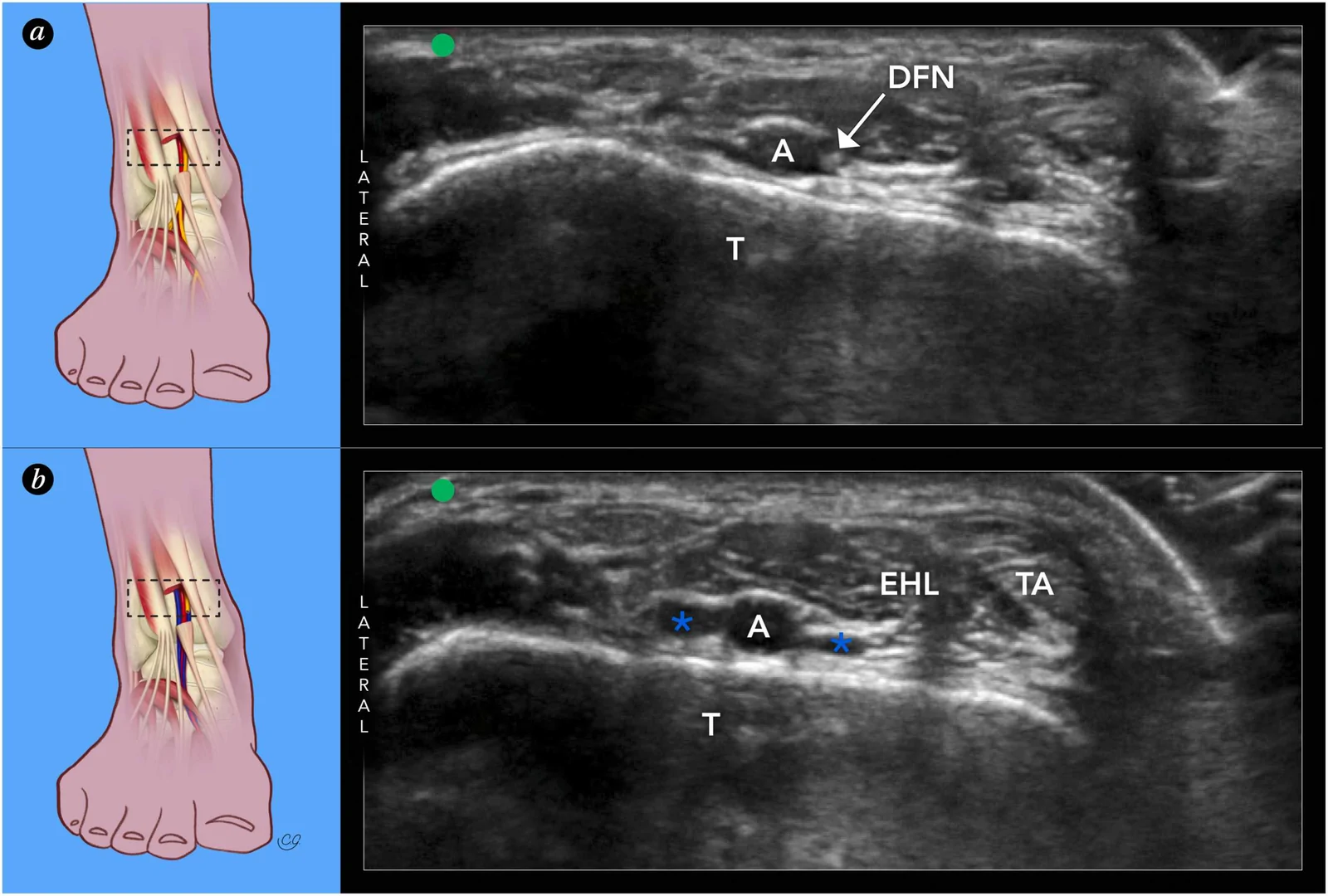
(a) Ultrasound image and probe position for the deep fibular nerve block. The surrounding veins and the dorsalis pedis artery are visualized after release of probe pressure in (b). Please note that the neurovascular bundle is typically lateral to the tibialis anterior (TA) tendon and deep to the extensor hallucis longus (EHL). The latter structure can be easily identified by plantarflexion of the big toe. DPA: dorsalis pedis artery, DFN: deep peroneal nerve, T: tibia, *: veins. Reproduced with permission from Girón Arango Medicine Professional Corporation.

The deep fibular nerve (DFN) is a terminal branch of the common fibular (peroneal) nerve (CFN). It is a mixed nerve that innervates the muscles of the anterior compartment of the leg, as well as the extensor digitorum brevis (EDB) and extensor hallucis brevis (EHB) muscles. The medial branch provides sensory innervation to the first interdigital space, and the lateral branch may also contribute sensory innervation to the sinus tarsi region.^11^, ^12^ After branching from the CFN, the DFN travels between the peroneus longus muscle and the fibula. It then pierces the transverse crural intermuscular septum and enters the anterior compartment of the leg. Continuing its downward course, the DFN enters the extensor retinaculum at the ankle joint level (Fig. 3) ^12^ The DFN is commonly identified approximately five fingerbreadths proximal to the ankle joint, lying on the interosseous membrane in close association with the anterior tibial (dorsalis pedis) artery. At the ankle, the nerve is typically lateral to the artery, though its relationship may vary proximally.^13^ The artery lies deep to the extensor hallucis longus (EHL) tendon, with the nerve positioned between the deep fascia and periosteum (Fig. 4). Adjacent veins are frequently present and should be recognized during scanning. This can be achieved by releasing pressure on the probe (Fig. 4, Video S3 and S4). Distal to the ankle joint, the nerve further divides into lateral and medial branches. The lateral branch innervates the EDB and EHB muscles, while the medial branch provides sensory innervation to the first interdigital space.^11^, ^12^ The motor branches to the muscles of the anterior compartment are given off more proximally and are therefore spared with this approach**.**

### Superficial fibular (peroneal) nerve

**Fig. 5:**
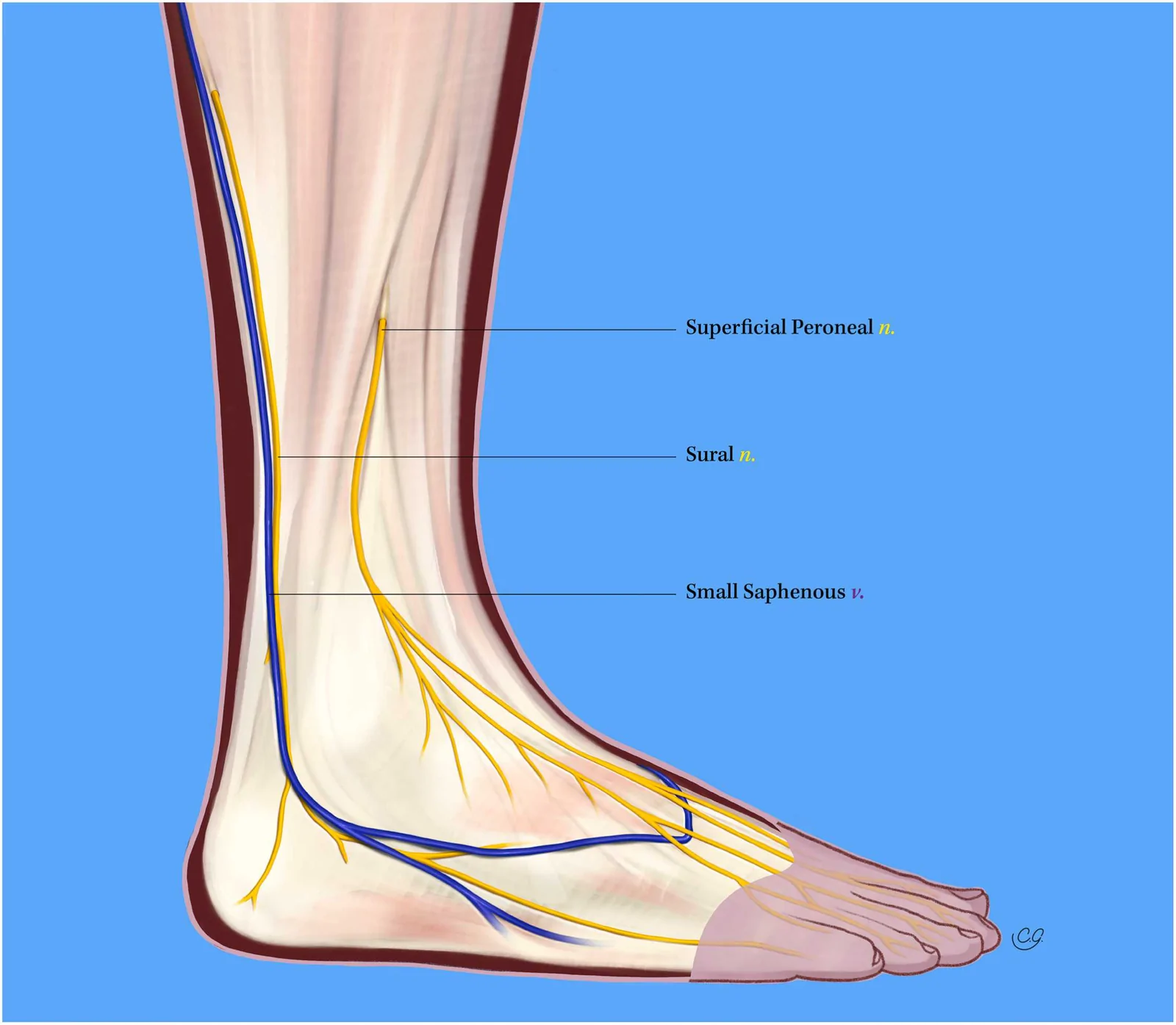
Illustration of the lateral aspect of the leg, highlighting the superficial fibular nerve (SPN). Please note that this illustration represents the typical course of the SFN, but variations in the nerve's course are possible and are described further in the article. n: nerve, v: vein. Reproduced with permission from Girón Arango Medicine Professional Corporation.

**Fig. 6:**
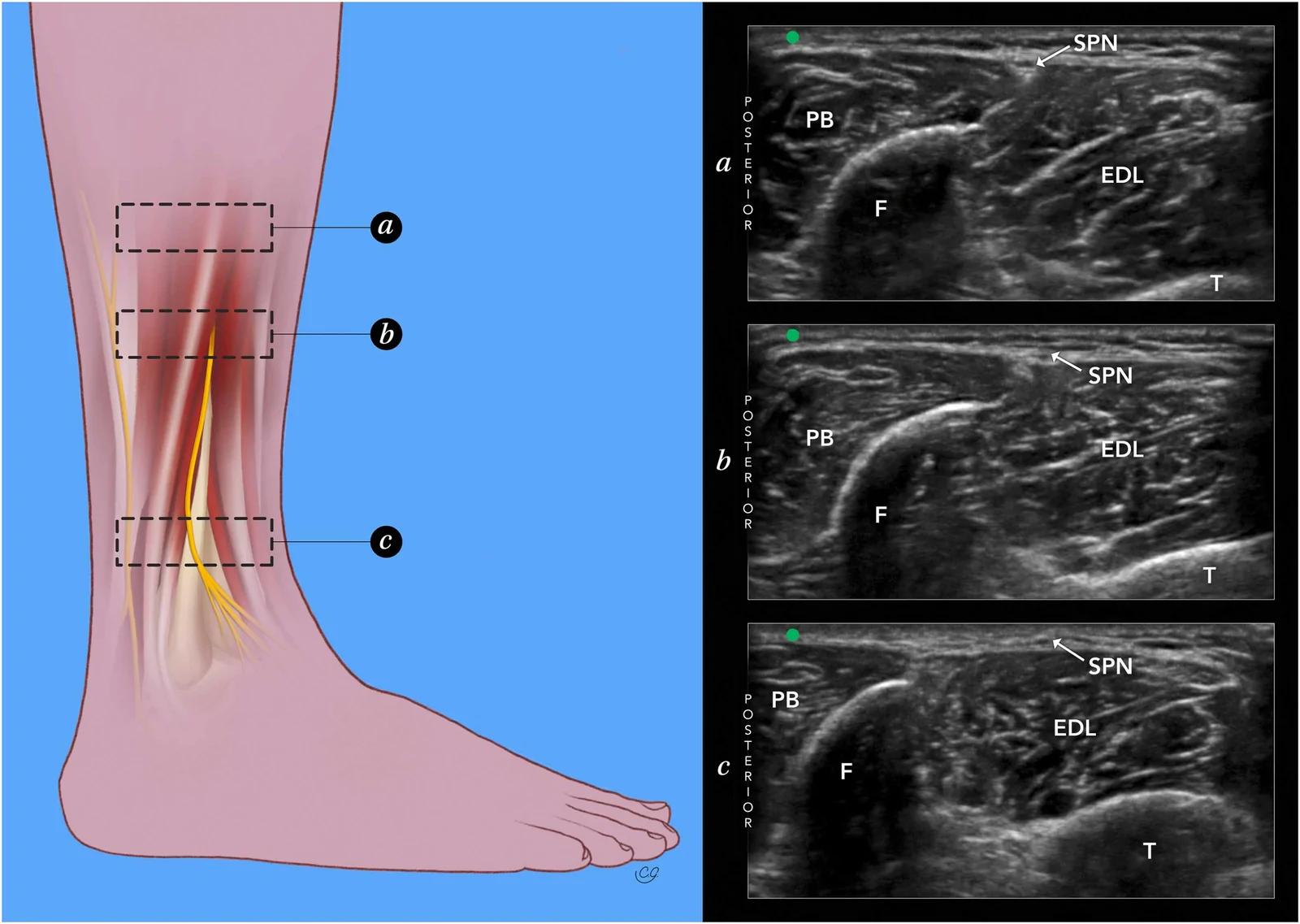
Ultrasound image and probe position for the superficial fibular nerve block, illustrating three key scanning landmarks for nerve identification. (a) Proximal image of the superficial fibular nerve deep to the fascia. (b) Image of the superficial fibular nerve (SFN) within the fascia. (c) Distal image of the superficial fibular nerve in the subcutaneous plane. We propose a systematic scanning method from proximal to distal for two reasons: 1. the SFN is small and hypoechoic in the subcutaneous plane and can be very challenging to find if the initial scan is at the distal leg; 2. in some individuals, the two terminal branches, the medial and intermediate dorsal cutaneous branch nerves, can bifurcate high up as they emerge superficial to the crural fascia, and a distal scan may miss one of the branches. EDL: extensor digitorum longus muscle, F: fibula, PB: peroneus brevis muscle, SPN: superficial peroneal nerve, T: tibia. Reproduced with permission from Girón Arango Medicine Professional Corporation.

The superficial fibular nerve (SFN) arises from the CFN and innervates most of the foot’s dorsum, as well as the peroneus longus (PL) and brevis (PB) muscles.^8^ Both motor branches arise proximally, at a variable distance from the fibular head, ranging from 2 to 10 cm.^14^ In the proximal leg, the nerve lies deep to the crural fascia and passes through the intermuscular septum between the extensor digitorum longus (EDL) and PL muscles. At approximately the distal third of the leg, between 19 and 28 cm from the tibial head, the nerve pierces the muscular fascia, becomes subcutaneous, and then divides into terminal sensory branches near the ankle (Fig. 5).^14^, ^15^ Owing to its superficial location and early branching pattern, direct visualization can be challenging, particularly in the presence of surgical scars or altered anatomy. Dynamic scanning allows identification of the nerve as it pierces the muscular fascia and facilitates its localization (Fig. 6, Video S5 and S6). The nerve is most often located anterior to the fibula, and careful scanning is required to avoid missing divided branches.

### Saphenous nerve

The saphenous nerve (SN) is a purely sensory nerve and the only component of the ankle block that originates from the lumbar plexus, arising from the femoral nerve.^8^, ^16^ Distal to the knee, it courses posterior to the saphenous vein toward the medial aspect of the ankle and foot, maintaining a close anatomical relationship with the vein. Approximately 3 cm proximal to the tip of the medial malleolus, the SN divides into anterior and posterior branches relative to the saphenous vein (Fig. 1).^17^, ^18^ The posterior branch terminates at the level of the medial malleolus, whereas the anterior branch continues over the anteromedial ankle to supply the medial ankle and foot.^19^

**Fig. 7:**
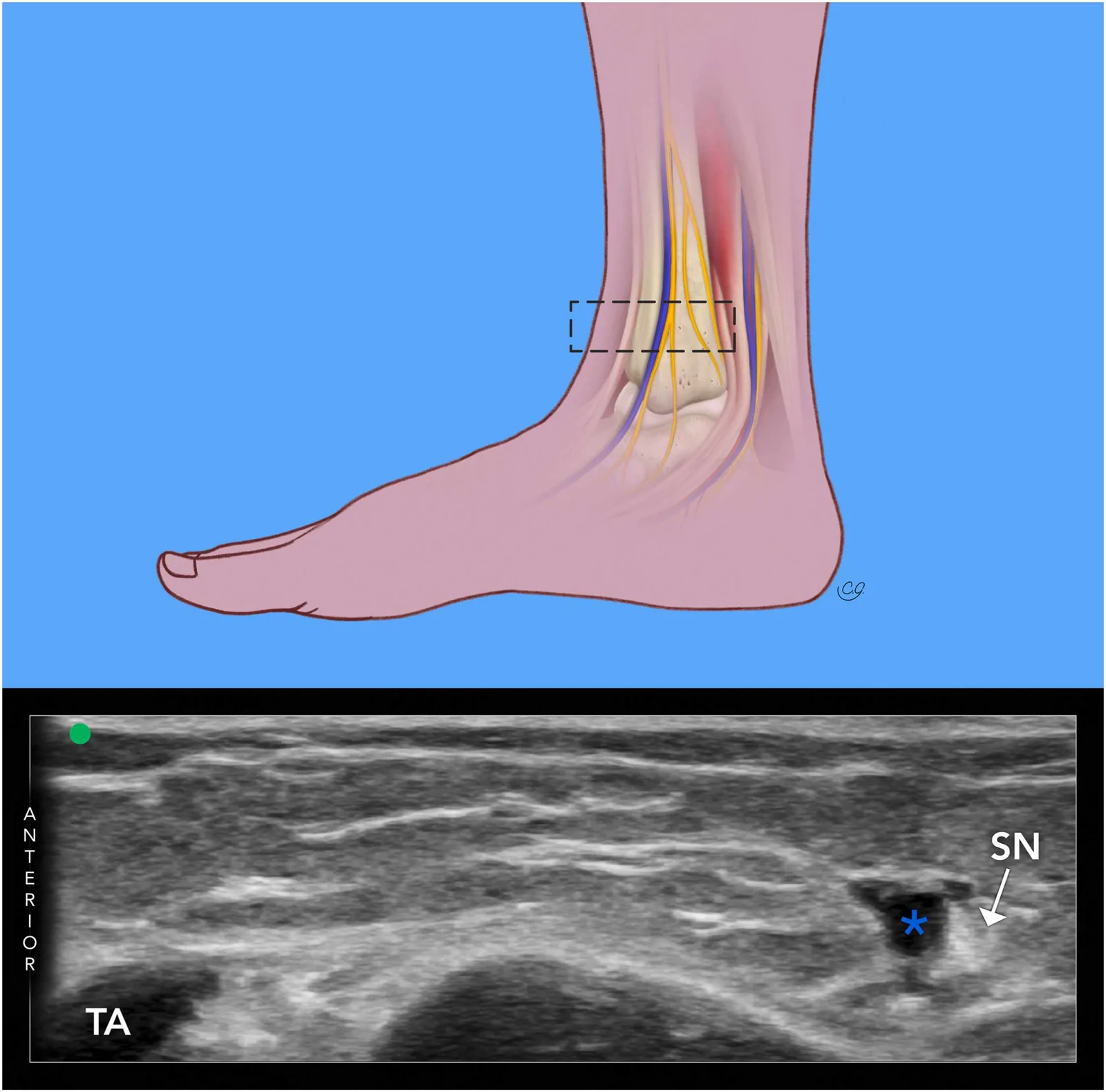
Ultrasound image and probe position for identification of the saphenous nerve. Please note that the anterior branches of the saphenous nerve can be visualized in the subcutaneous plane between the tibialis anterior tendon and the midpoint of the medial malleolus. Only the posterior branches are missed, but they do not innervate the forefoot and midfoot. *: saphenous vein, SN: saphenous nerve, TA: tibialis anterior tendon. Reproduced with permission from Girón Arango Medicine Professional Corporation.

At the ankle level, the SN is consistently located in a superficial subcutaneous plane between the tibialis anterior tendon and the medial malleolus (Fig. 7). Effective blockade is therefore typically achieved with subcutaneous local anesthetic infiltration under ultrasound guidance around the great saphenous vein, rather than with targeted perineural injection (Video S7). When multiple veins are present in this plane, subcutaneous infiltration between the tibialis anterior tendon and the medial malleolus provides adequate coverage. At this level, the posterior branch may be missed; however, it supplies only the medial malleolus and is not required for foot analgesia.

### Sural nerve

**Fig. 8:**
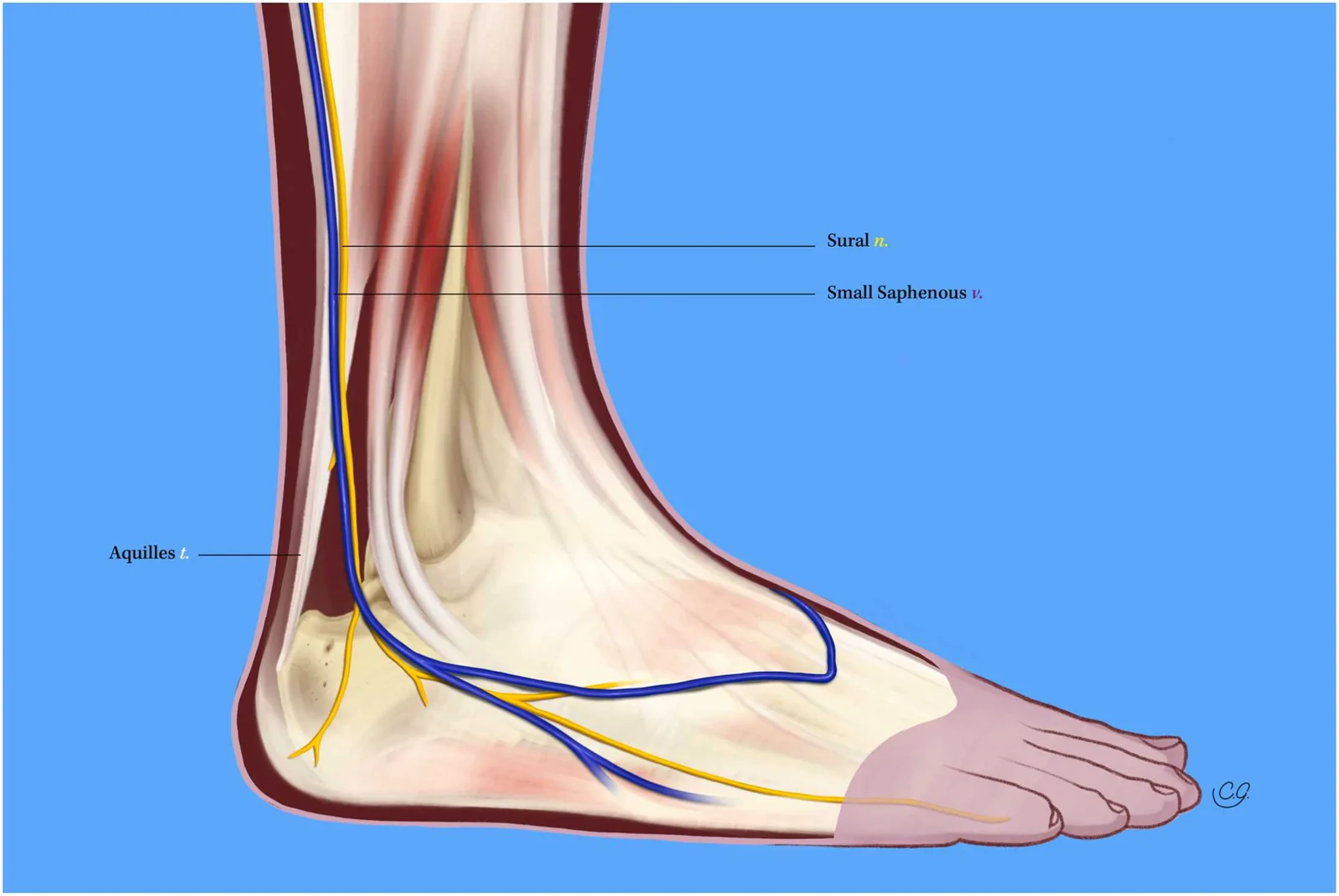
Illustration of the lateral aspect of the leg, highlighting the sural nerve. Please note that the fascia overlying the sural nerve was removed. n: nerve, t: tendon, v: vein. Reproduced with permission from Girón Arango Medicine Professional Corporation.

**Fig. 9:**
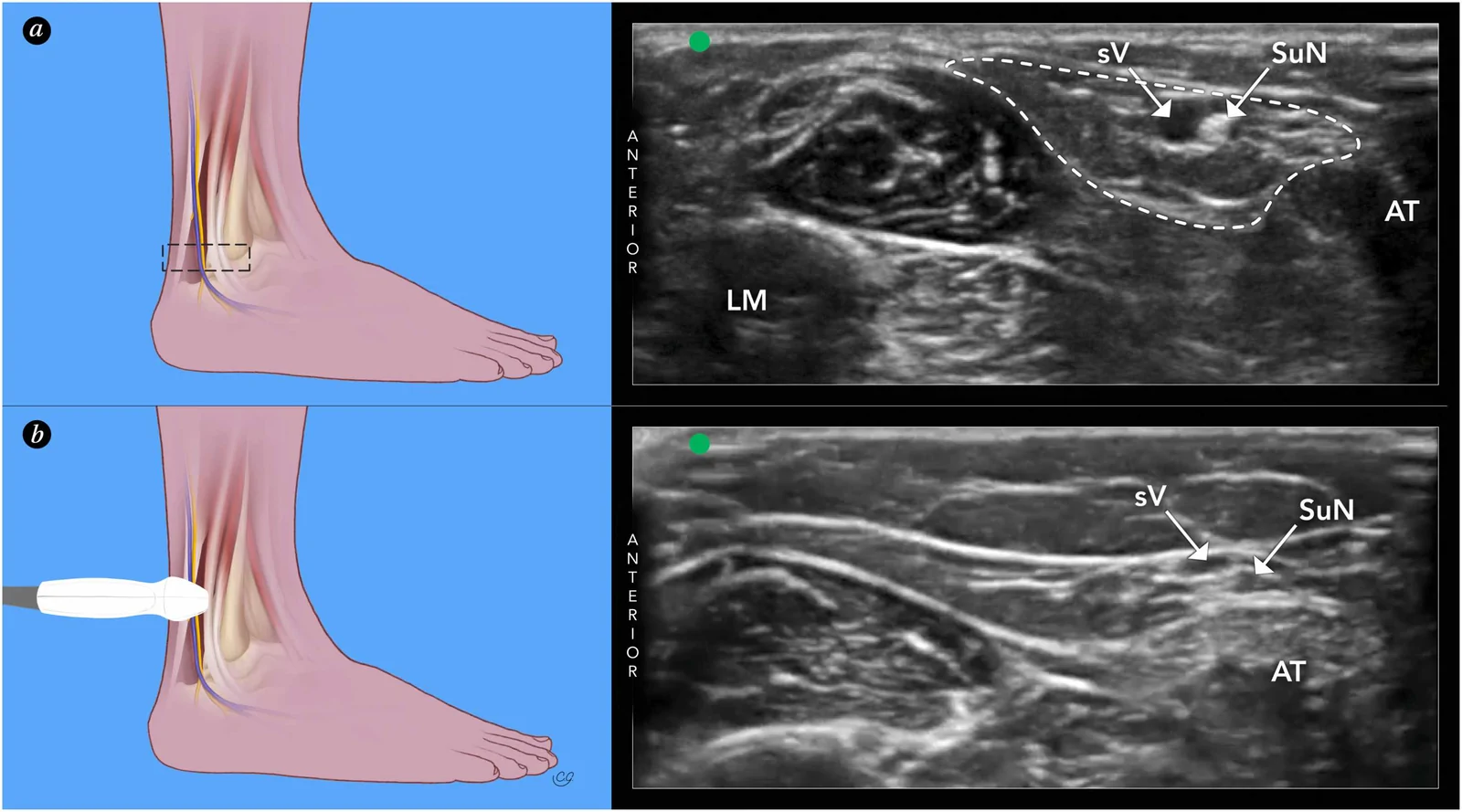
Ultrasound image and probe positions for the sural nerve block. (a) In the distal view, the probe is placed between the lateral malleolus and the Achilles tendon, where the sural nerve is identified within the fascial expansion (arrowheads) adjacent to the lesser saphenous vein. (b) To obtain a clearer view of the sural nerve, the probe is moved proximally and posteriorly. The sural nerve is seen closer and superficial to the Achilles tendon. LM: lateral malleolus, sV: lesser saphenous vein, SuN: sural nerve. Reproduced with permission from Girón Arango Medicine Professional Corporation.

The sural nerve is formed by contributions from the tibial and common peroneal nerves, joining posterior to the soleus muscle and descending along the posterolateral aspect of the calf.^8^ It provides sensory innervation to the lateral aspect of the foot’s dorsum.^20^ At the ankle, the sural nerve is commonly identified approximately five fingerbreadths proximal to the lateral malleolus, coursing between the Achilles tendon and the peroneal tendons, and is consistently associated with the lesser saphenous vein (Fig. 8, Video S8). Visualization may be facilitated by tracing the nerve proximally toward the Achilles tendon, particularly in patients without an accompanying vein or with prior surgical intervention (Fig. 9, Video S9).

## Popliteal sciatic nerve block

**Fig. 10:**
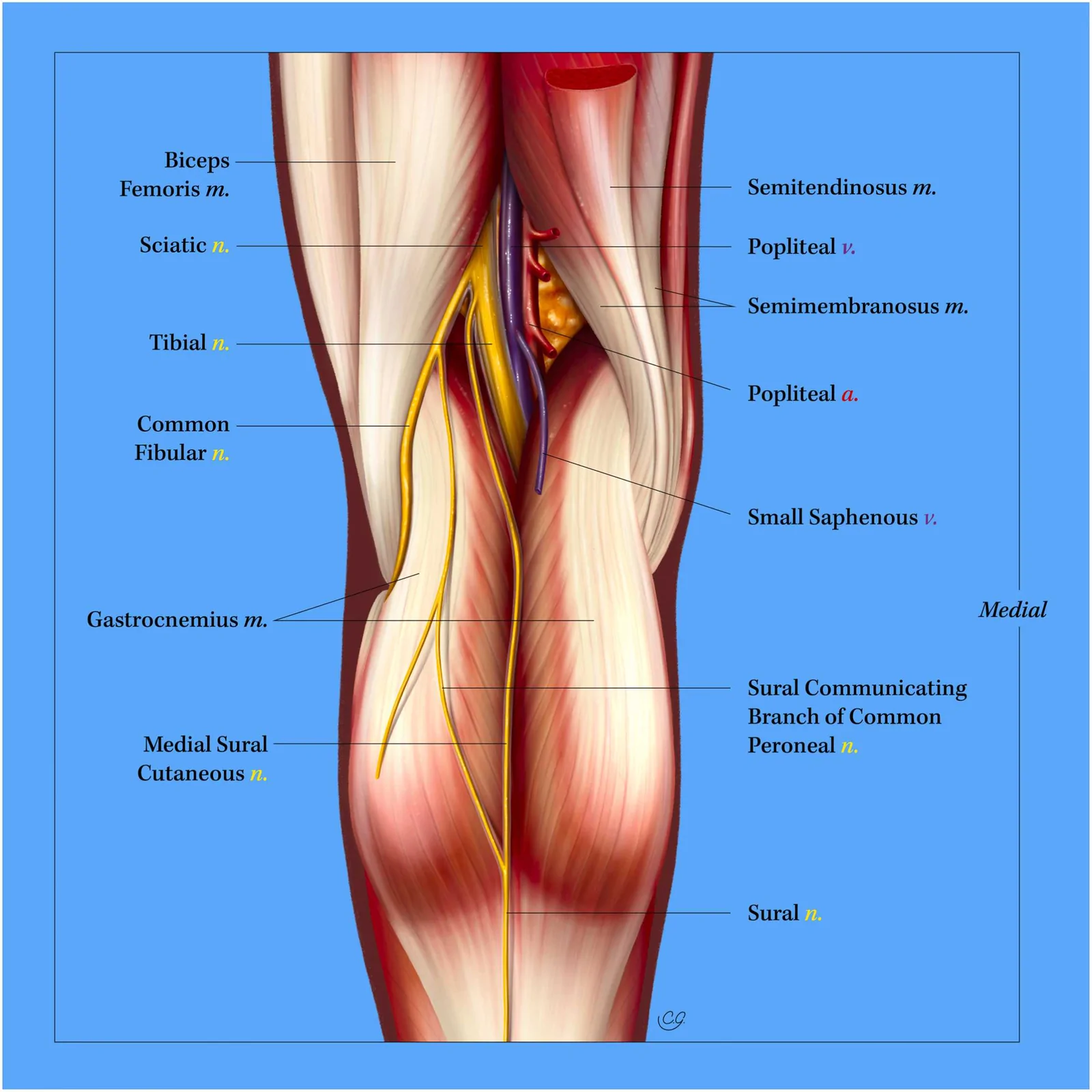
Illustration of the popliteal fossa anatomy. a: artery, n: nerve, v: vein. Reproduced with permission from Girón Arango Medicine Professional Corporation.

The sciatic nerve is the largest branch of the lumbosacral plexus, arising from the L4–L5 and S1–S3 spinal nerves. It comprises two distinct nerves—the tibial nerve (TN) and the common fibular nerve (CFN)—which travel together within a common paraneural sheath. Proximal to the popliteal fossa, the sciatic nerve divides into the TN and CFN (Fig. 10, Video S10). The level of sciatic nerve bifurcation shows substantial anatomical variability. Although the sciatic nerve most commonly divides into the tibial and common fibular nerves in the distal thigh or at the superior aspect of the popliteal fossa, cadaveric studies have shown that division may occur at more proximal levels, including the pelvis, gluteal region, or thigh. Recent evidence suggests that approximately one-third of sciatic nerves deviate from the typical pattern, with the popliteal fossa the most common site of anatomical variation.^21^ Awareness of these variations is important during ultrasound-guided regional anesthesia, as separate tibial and common fibular nerve components may be encountered during scanning and may require individual identification to ensure complete sensory blockade.

Blockade of the sciatic nerve at the popliteal fossa level provides analgesia for ankle procedures and may serve as an anesthetic technique when combined with a saphenous nerve block at the adductor canal to ensure medial leg coverage.^22^

For popliteal sciatic nerve blocks, a high-frequency probe usually provides adequate depth, but in patients with increased soft-tissue depth, lower-frequency linear or curvilinear transducers may be required to optimize penetration.

**Fig. 11:**
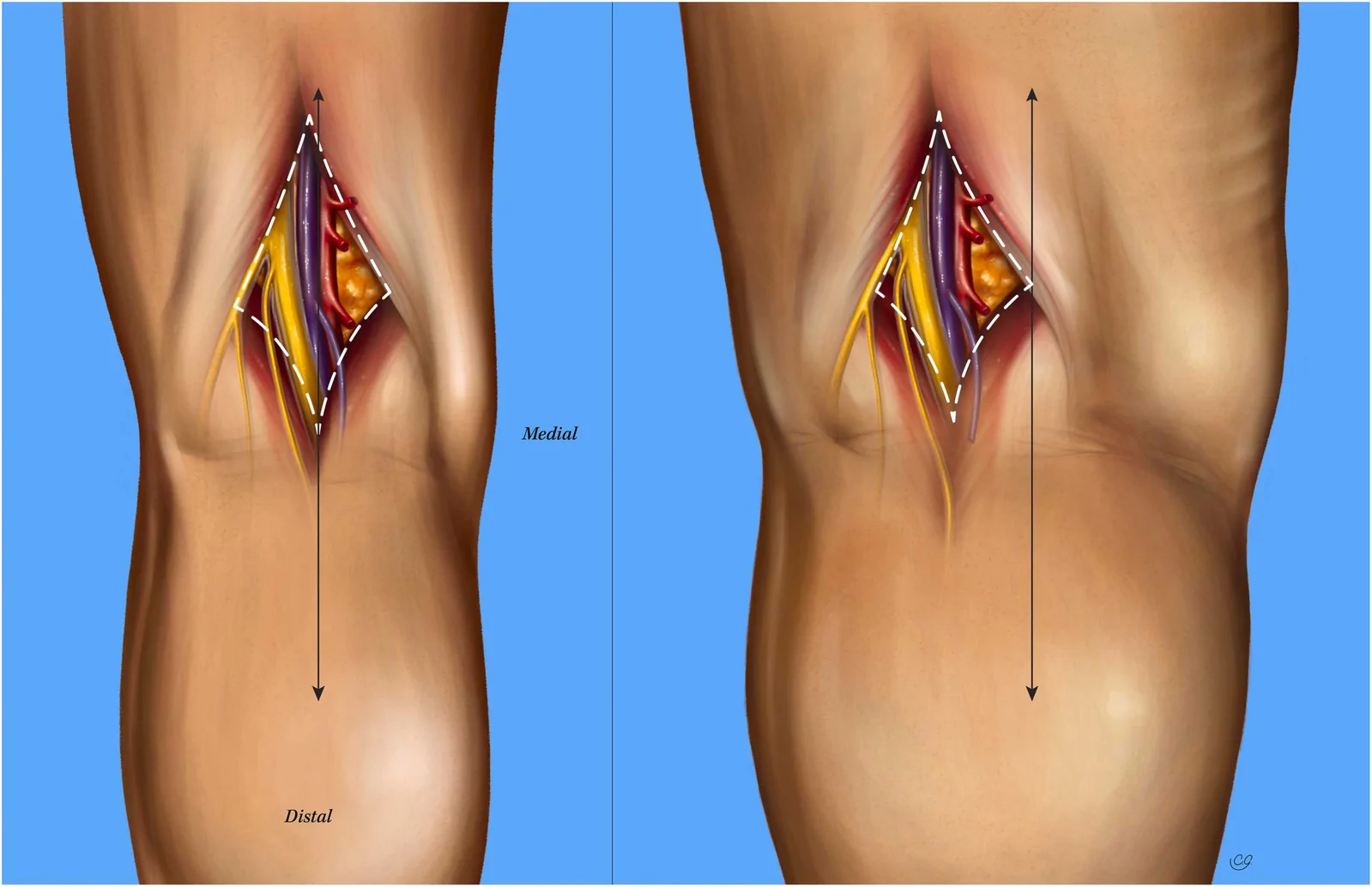
Illustrations of the popliteal fossa. Left: expected location of the popliteal fossa in an individual without significant adiposity. The midline of the fossa can be readily estimated from the surface landmark. Right: medial adiposity displaces the popliteal fossa laterally. As a result, the anatomic midline of the popliteal fossa deviates substantially from the thigh's midline and the surface landmark. Reproduced with permission from Girón Arango Medicine Professional Corporation.

**Fig. 12:**
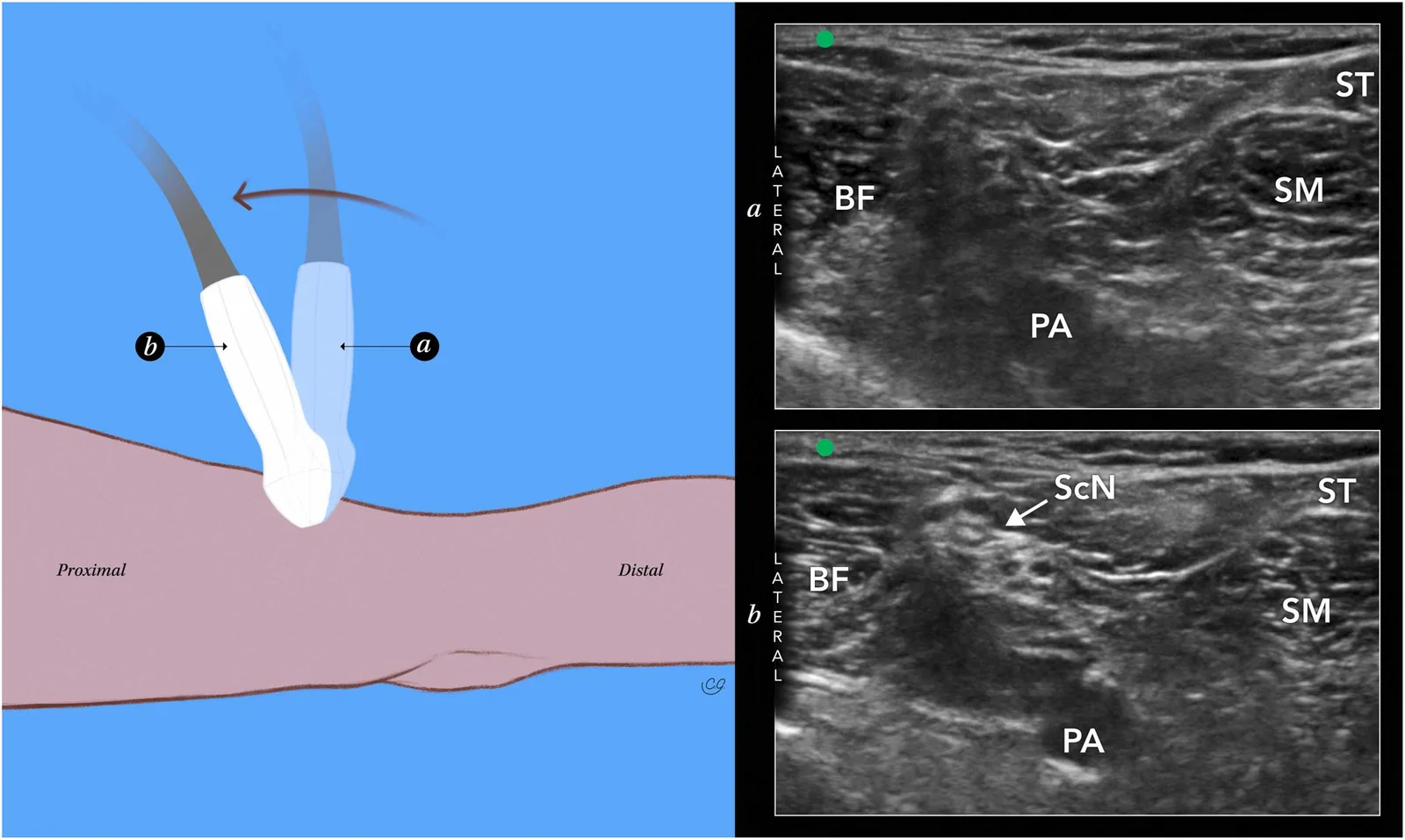
Illustration and ultrasound images showing how probe tilting optimizes sciatic nerve visualization. With the probe perpendicular to the leg (a), visualization of the sciatic nerve may be limited by anisotropy. Tilting the probe caudally (b) improves nerve echogenicity and visualization. BF: biceps femoris muscle, PA: popliteal artery, SM: semimembranosus muscle, ScN: sciatic nerve, ST: semitendinosus muscle. Reproduced with permission from Girón Arango Medicine Professional Corporation.

**Fig. 13:**
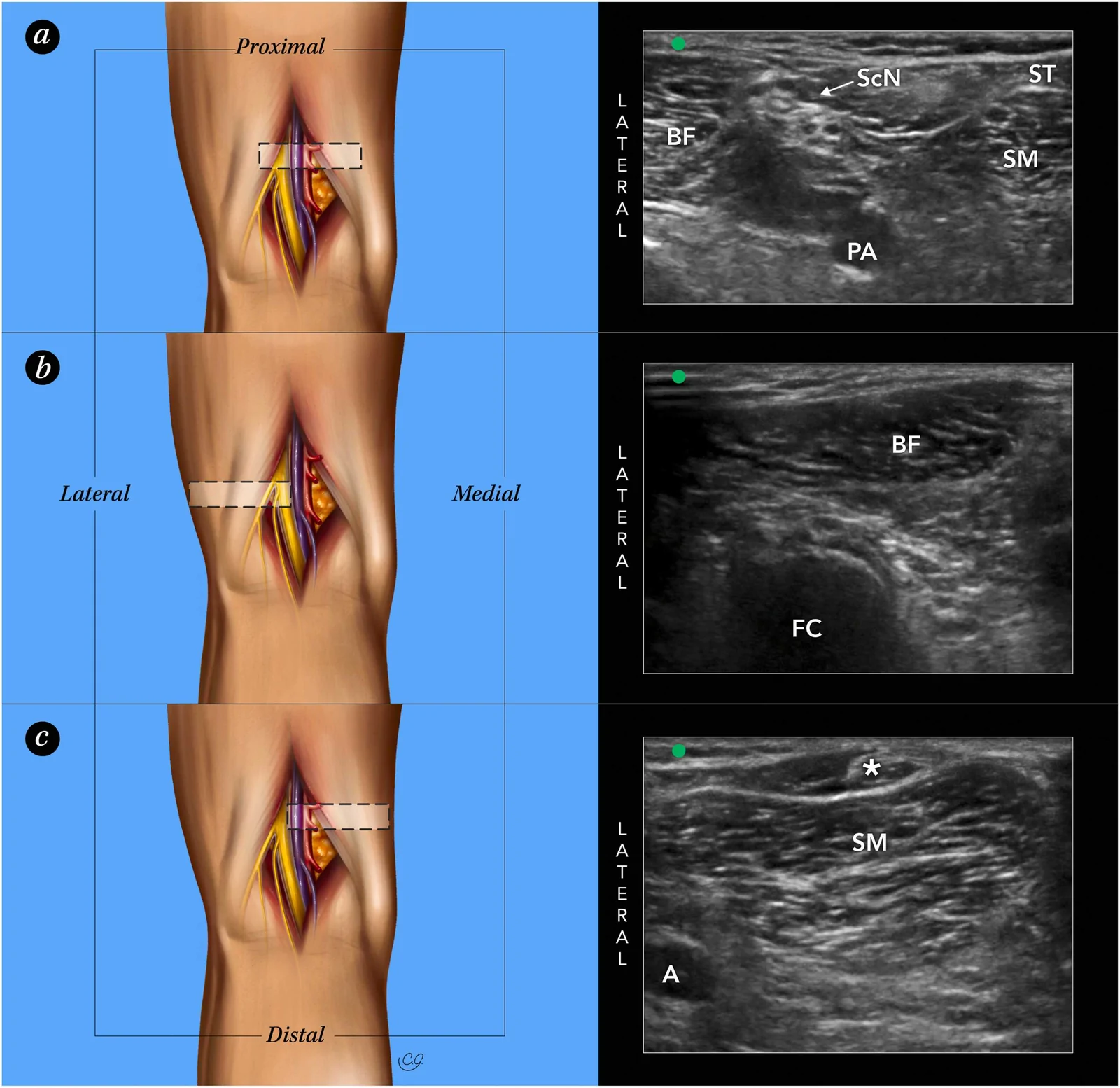
Ultrasound images and probe positions for the popliteal sciatic nerve block. Three probe positions are illustrated: (a) placement at the level of the popliteal fossa, identifying the sciatic nerve and its tibial and common fibular components; (b) lateral placement, identifying the biceps femoris muscle on the posterior aspect of the femoral condyle; and (c) medial placement, identifying the semimembranosus and semitendinosus muscles, producing the characteristic “cherry-on-the-cake” appearance. A: popliteal artery, BF: biceps femoris muscle, FC: femoral condyle, PA: popliteal artery, SM: semimembranosus muscle, ScN: sciatic nerve, *: semitendinosus tendon. Reproduced with permission from Girón Arango Medicine Professional Corporation.

Accurate identification of the sciatic nerve at the mid-popliteal fossa is challenging for two reasons. In patients with very high body mass index (BMI), there can be a large discrepancy between the anatomic midline of the popliteal fossa and the surface landmark midline (Fig. 11). To improve visualization, caudal probe tilting is typically required to enhance anisotropy and distinguish neural structures from surrounding soft tissue (Fig. 12). In challenging cases when the sciatic nerve cannot be found in the popliteal fossa, we propose first identifying the medial and lateral borders of the popliteal fossa (Fig. 13a). The lateral border is marked by the biceps femoris, which attaches to the lateral side of the fibular head. It can be identified at the distal tibial level on the posterior aspect of the femoral condyle (Fig. 13b). The medial border is marked by the semitendinosus and semimembranosus. At the distal tibial level, these two muscles appear as a “cherry on the cake,” with the semitendinosus lying over the semimembranosus (Fig. 13c). The midline of the popliteal fossa can be easily identified even in patients with high BMI. Once the midline is identified, the sciatic nerve can be found by optimizing the probe tilt (Fig. 12). The out-of-plane technique for the popliteal sciatic nerve block is demonstrated in Video S11.

To facilitate reproducibility and standardization of the techniques described, Table 1 summarizes the recommended patient positioning, transducer orientation, and typical local anesthetic volume for each block covered in this pictorial essay. These recommendations are intended to complement the accompanying instructional videos and provide readers with a quick reference to the key procedural elements of each technique.

**Table 1:** Patient position, ultrasound transducer placement, and suggested local anesthetic volume for lower limb nerve blocks.

**Block**	**Patient position**	**Transducer orientation**	**Recommended local anesthetic volume**
Tibial nerve	Supine with the hip externally rotated and knee slightly flexed	Transverse at the medial aspect of the leg posterior to the medial malleolus	8–10 mL
Deep fibular nerve	Foot in neutral position with the knee flexed	Transverse over the anterior aspect of the leg cephalad to the ankle joint at the level of the dorsalis pedis artery.	5 mL
Superficial fibular nerve	Supine with the knee flexed and the hip internally rotated.	Transverse over the anterolateral distal leg	8–10 mL
Saphenous nerve	Supine with the leg externally rotated	Transverse at the medial aspect of the leg, anterior to the medial malleolus	5 mL
Sural nerve	Supine with the leg internally rotated	Transverse at the posterolateral aspect of the leg, posterior to the lateral malleolus	5 mL
Popliteal sciatic nerve	Prone or lateral decubitus	Transverse at the popliteal fossa	20 mL

## Conclusions

Ultrasound-guided ankle and popliteal sciatic nerve blocks provide effective anesthesia and analgesia for foot and ankle surgery, respectively, when performed with a thorough understanding of relevant anatomy and sonoanatomy. Awareness of anatomical variations and consistent use of ultrasound landmarks are essential to avoid incomplete sensory coverage. By illustrating the sonoanatomy of the sciatic nerve and its distal branches, this pictorial essay aims to support anesthesiologists in developing a systematic approach to image acquisition and nerve identification, thereby improving block reliability and clinical outcomes.

## CRediT authorship contribution statement

**Philip Peng:** Conceptualization; Supervision; Visualization; Validation; Writing – review & editing. **Laura Giron Arango:** Conceptualization; Writing – original draft; Writing – review & editing. **Carlos Andrés Guzmán-Hernández:** Writing – review & editing; Visualization. All authors have read and agreed to the published version of the manuscript.

## Consent for publication

Not applicable.

## Ethical statement

Not applicable.

## Funding

The authors have no sources of funding to declare for this manuscript.

## Declaration of competing interest

Carlos Andrés Guzmán-Hernández receives non-clinical time support from the Department of Anesthesia and Pain Management, Toronto Western Hospital, University Health Network. Philip Peng provides equipment support from Shenzhen Wisonic Medical Technology Co., Ltd., Shenzhen, China.

## Data Availability

No datasets were generated and/or analyzed for this manuscript.

## Appendix A Supplementary material

Video S1. Ultrasound video showing contraction and movement of the flexor hallucis longus tendon during active or passive hallux motion. The tibial neurovascular bundle, including the tibial nerve, artery, and veins, is visualized superficial to the tendon and its fascia. Reproduced with permission from Girón Arango Medicine Professional Corporation

**Video S2.** Video of a tibial nerve block. Patient position: Supine with the hip externally rotated and the knee slightly flexed. Transducer orientation: Transverse at the medial aspect of the leg, posterior to the medial malleolus. Needle approach: In-plane, anterior-to-posterior. TN: Tibial nerve. Reproduced with permission from Girón Arango Medicine Professional Corporation

**Video S3.** Dynamic compression with the ultrasound transducer shows collapse of the accompanying veins while the tibial artery remains visible. Arrows: Veins. Reproduced with permission from Girón Arango Medicine Professional Corporation

**Video S4.** Video of a deep fibular nerve block. Patient position: Supine with the foot in a neutral position and the knee flexed. Transducer orientation: Transverse over the anterior aspect of the leg cephalad to the ankle joint at the level of the dorsalis pedis artery. Needle approach: In-plane, lateral-to-medial. DFN: Deep fibular nerve. Reproduced with permission from Girón Arango Medicine Professional Corporation

**Video S5.** Dynamic proximal-to-distal scanning shows the superficial fibular nerve as it pierces the crural fascia and enters the subcutaneous plane. SFN: Superficial fibular nerve. Reproduced with permission from Girón Arango Medicine Professional Corporation

**Video S6.** Video of a superficial fibular nerve block. Patient position: Supine with the knee flexed and the hip internally rotated. Transducer orientation: Transverse over the distal anterolateral aspect of the leg. Needle approach: In-plane, posterior-to-anterior. SFN: Superficial fibular nerve. Reproduced with permission from Girón Arango Medicine Professional Corporation

**Video S7.** Video of a saphenous nerve block. Patient position: Supine with the hip externally rotated. Transducer orientation: Transverse at the medial aspecto of the leg, anterior to the medial malleolus**.** Needle approach: In-plane, anterior-to-posterior. SV: Saphenous vein. Reproduced with permission from Girón Arango Medicine Professional Corporation

**Video S8.** Video of a sural nerve block. Patient position: Supine with the hip internally rotated. Transducer orientation: Transverse at the posterolateral distal aspect of the leg, posterior to the lateral malleolus**.** Needle approach: In-plane, anterior-to-posterior. SuN: Sural nerve. Video of an in-plane saphenous nerve block. SV: Saphenous vein. Reproduced with permission from Girón Arango Medicine Professional Corporation

**Video S9.** Dynamic distal-to-proximal scanning demonstrates the changing relationship between the sural nerve and the Achilles tendon, with the nerve becoming progressively more posterior and closer to the tendon. SuN: Sural Nerve. Reproduced with permission from Girón Arango Medicine Professional Corporation

**Video S10.** Dynamic proximal-to-distal scanning of the sciatic nerve demonstrates the bifurcation into the fibular and tibial components of the sciatic nerve. CFN: Common fibular nerve, TN: Tibial nerve. Reproduced with permission from Girón Arango Medicine Professional Corporation

**Video S11.** Video of a popliteal sciatic nerve block. Position: Prone. Transducer orientation: Transverse at the popliteal fossa. Needle approach: Out-of-plane. CFN: Common fibular nerve; TN: Tibial nerve. Reproduced with permission from Girón Arango Medicine Professional Corporation

## Footnotes

Appendix A Supplementary data associated with this article can be found in the online version at doi:10.1016/j.jatmed.2026.07.003.
